# The association of Curcuma and Scutellaria plant extracts improves laying hen thermal tolerance and egg oxidative stability and quality under heat stress conditions

**DOI:** 10.3389/fvets.2022.957847

**Published:** 2022-08-03

**Authors:** Ilias Giannenas, Panagiotis Sakkas, Georgios A. Papadopoulos, Ioannis Mitsopoulos, Ioanna Stylianaki, Stella Dokou, Vasileios Tsiouris, Theodora Papagrigoriou, Marina Panheleux, Fabrice Robert, Vasileios A. Bampidis

**Affiliations:** ^1^Laboratory of Nutrition, Faculty of Veterinary Medicine, Aristotle University, Thessaloniki, Greece; ^2^CCPA Group, Janzé, France; ^3^Laboratory of Animal Husbandry, Faculty of Veterinary Medicine, Aristotle University, Thessaloniki, Greece; ^4^Division of Animal Science, Faculty of Agriculture, International Hellenic University, Thessaloniki, Greece; ^5^Laboratory of Pathology, Faculty of Veterinary Medicine, Aristotle University, Thessaloniki, Greece; ^6^Unit of Avian Medicine, Clinic of Farm Animals, Aristotle University of Thessaloniki, Thessaloniki, Greece; ^7^Laboratory of Pharmacognosy, Faculty of Health Sciences, School of Pharmacy, Aristotle University of Thessaloniki, Thessaloniki, Greece

**Keywords:** laying hen, heat stress, Curcuma and Scutellaria, egg quality, liver evaluation

## Abstract

Chronic exposure to high ambient temperatures is detrimental to laying hen performance and egg quality. Plant secondary metabolites may alleviate effects, partly due to their antioxidant activities. Herein, we investigated the effects of dietary supplementation with a phytonutrient solution (PHYTO) consisting of a plant extract combination of *Scutellaria baicalensis* and *Curcuma longa* on young layers (25–32 wk of age) raised under naturally elevated temperature conditions. Four hundred, 24-wk-old Lohmann hens were allocated in 50 cages and, after a week of adaptation, were offered a diet either containing 2 g/kg of PHYTO or not, for 8 wk. Hen BW was measured at the beginning and end of the trial, and egg production and feed intake were recorded weekly. At week 32, four eggs per cage were collected to determine egg quality characteristics as well as the rate of lipid and albumen oxidation in fresh eggs. At the end of the trial, two hens per cage were blood sampled for assessment of biochemical markers, one of which was euthanized for histopathological evaluation of the liver and intestine and assessment of intestinal histomorphometry. The herbal mixture supplementation significantly increased egg production rate at weeks 28 and 29 and for the overall production period, and feed efficiency at weeks 26–29. In addition, the degree of liver necrosis and microvascular thrombosis was lower (*P* < 0.05) whereas intestinal villosity was greater in duodenal and jejunal segments (*P* < 0.05) in the PHYTO compared to the control group. Supplementation also reduced (*P* < 0.05) blood concentrations of corticosterone, alanine aminotransferase activity, and TBARS, and a reduction in catalase activity was observed. Egg quality characteristics were not affected, except for eggshell thickness, egg diameter, and eggshell breaking strength that were superior in the PHYTO group (*P* < 0.05). PHYTO supplementation significantly improved egg lipid oxidation status of fresh eggs. In conclusion, supplementation with PHYTO improved laying hen productivity and egg quality, which was associated with an improvement in laying hen thermotolerance.

## Introduction

In high-temperature areas, the occurrence of heat stress (HS) is one of the most important stressors for poultry, causing extensive economic losses, while challenging their health and welfare (1). Moreover, the effects of HS are expected to become more prominent due to climate change, which drives an increase in global temperatures characterized by heat waves of increasing intensity, duration, and frequency (2). Laying hens are particularly vulnerable to HS as they have a limited capacity to maintain the homeostasis of body temperature due to their lack of sweat glands and their excessive feather coverage (3), while they have a long production cycle (50–70 weeks) and are thus susceptible to its long-term effects (4).

When faced with HS, a culmination of behavioral, hormonal, immunological, physiological, metabolic, and biochemical changes leads to reductions in average daily feed intake (ADFI), egg production, feed efficiency, and egg quality (4, 5). A meta-analysis has shown that ambient temperatures above 24°C hens may impair performance and lead to production of eggs of inferior quality (5). Prevailing temperatures in the Mediterranean basin rest above these estimates for extended periods of time. Furthermore, poultry facilities are often not equipped with modern ventilation technologies to efficiently control in-house temperatures (6, 7).

One of the hallmarks of HS is the occurrence of oxidative stress as it directly increases mitochondrial energy generation causing an imbalance between pro-oxidant and antioxidant systems, defined by the presence of reactive species (RS), in excess of the available antioxidant capacity of animal cells (8). Increased production of RS leads to an impairment of mitochondrial function and damages proteins, lipids, and DNA (9, 10) Importantly, the shift of visceral blood flow toward the peripheral circulation during HS to facilitate heat dissipation induces hypoxic conditions in the intestine and renders it particularly susceptible to oxidative stress. Oxidative damage is not limited to the gastrointestinal tract but can also occur in multiple organs (11) and may lead to acute liver injury (12). These effects may be reflected in blood and in egg yolk or albumen by increased concentration of lipid and protein oxidation products.

Certain plant secondary metabolites (PSMs) contained in plant extracts may mitigate the adverse effects of HS on laying hen performance. Recent studies have demonstrated that dietary supplementation with curcuminoids contained in *Curcuma longa* (CUR) improved laying hen thermotolerance by upregulating antioxidant defenses (13), inhibiting the pro-inflammatory cytokine production and improving humoral immunity, while increasing steroidogenesis (14) and exhibiting hepatoprotective effects (13). Furthermore, curcumin supplementation reduced lipid peroxidation levels in egg yolk of laying hens raised in thermoneutral conditions (15). On the contrary, *Scutellaria baicalensis* (SCUT) and its active metabolites baicalin and baicalein have been shown to exhibit potent anti-inflammatory effects in poultry (16) and improve egg oxidative status (17), although research in laying hens exposed to HS is currently lacking. Previously, offering CUR extract alone was not enough to decrease gut inflammation induced by HS in broiler chicks. However, dietary supplementation with a phytonutrient solution (PHYTO) consisting of a mixture of CUR and SCUT plant extracts decreased gut inflammation induced by HS or S. Enteritidis infection (18), pointing toward synergistic effects between their active metabolites.

The aim of this study was to ascertain the effects of dietary supplementation of PHYTO in young laying hens around the peak of lay (25–32 weeks of age) raised under naturally elevated temperature conditions. We hypothesized that supplementation would lead to improved performance due to reduced liver and intestinal stress, as assessed by liver histopathological and intestinal histomorphometrical and histomorphological analysis, respectively, and effects would be associated with improved hen systemic antioxidant status, egg quality, and egg oxidation status.

## Materials and methods

### Animals, diets, and experimental design

The trial protocol was authorized by the Research Committee of Aristotle University, Thessaloniki, Greece (number 71553, 28.07.2020). Husbandry, euthanasia, experimental procedures, and biosecurity precautions were conducted in accordance with all welfare requirements described by Good Farming Practice Guidelines (Directive 2010/63/EC; Commission recommendation 2007/526/EC) and were approved by the Research Ethics Committee of the Aristotle University of Thessaloniki. Throughout the trial, birds were handled in compliance with local laws and regulations (19) and in accordance with the principles and guidelines for poultry welfare (20). Four hundred Lohmann Brown-Classic laying hens (24-week-old), kept at the poultry farm of the International Hellenic University (IHU, Sindos, Thessaloniki, Greece; 40°39′ N, 22°48′ E), were used in this study. Birds were housed in 50 replicate furnished cages (length 0.8 m; width 0.4 m; height 0.3 m) of eight hens each and were offered a commercial corn and soybean meal-based feed in mash form formulated to meet or exceed the requirements for nutrients and energy content for laying hens (21) (Table 1). From weeks 25 to 32, birds were either maintained in the same feed (Control) or offered the same diet supplemented with a commercial feed additive product (Phyto) consisting of an extract from *C. longa* (CUR) and hydrosoluble flavonoid extract from *S. baicalensis* (SCUT), added at the rate of 0.2 (%) diet replacing an equivalent amount of wheat bran, as described in a previously published study (18). The PHYTO product was pre-mixed with calcium carbonate as a carrier prior to its incorporation to the diets, and constituent plant extracts of CUR and SCUT were included at a 1:1 ratio. The principal bio-active metabolites of the extracts used are baicalin and curcumin for SCUT and CUR, respectively. The dose tested of the plant extract combination was based on the results of a previously published study where supplementation was effectively shown to decrease gut inflammation induced by heat stress or *S. enteritidis* infection, in broiler chicken (18). Experimental diets were formulated to meet or exceed the requirements for nutrients and energy content for laying hens (21). Birds had *ad libitum* access to feed and water. The lighting program was set at 16 h of continuous light per day.

**Table 1:** Ingredients and composition of the control layer diet.

**Ingredients**	**Composition (g/kg)**
Maize, grains	552.0
Wheat, grains	50.0
Soybean meal	240.0
Wheat bran	33.0
Soy oil	5.0
Limestone	95.0
Monocalcium phosphate	10.5
DL-Methionine	3.2
Lysine	1.3
Threonine	0.5
Valine	1.0
Sodium chloride, iodized	2.3
Sodium bicarbonate	2.2
Vitamin premix^a^	1.5
Trace-mineral premix^b^	1.5
Total	1000.0
**Calculated analysis** ^c^	**(g/kg)**
Dry matter	883.9
Crude protein	167
Ether extract	27
Crude fiber	33
Ash	84.1
Calcium	36.5
Phosphorus (total)	6.5
Metabolizable energy (MJ/kg)	11.6

a Supplying per kg feed: 4.82 mg all-trans retinol acetate, 62.5 μg cholecalciferol, 30 mg α-tocopheryl acetate, 2 mg menadione sodium bisulfite, 2 mg thiamine hydrochloride, 3 mg riboflavin, 4 mg pyridoxine hydrochloride, 0.02 mg cyanocobalamin, 20 mg niacin, 10 mg pantothenic acid, 1.0 mg folic acid, 0.07 mg biotin, 50 mg ascorbic acid, 300 mg choline chloride, and 40 mg carotenoids.

b Supplying per kg feed: 80 mg Zn, 40 mg Mn, 160 mg Fe, 70 mg Cu, 0.25 mg Co, 1 mg I, and 0.2 mg Se.

c According to NRC (21).

### Ambient conditions

**Figure 1:**
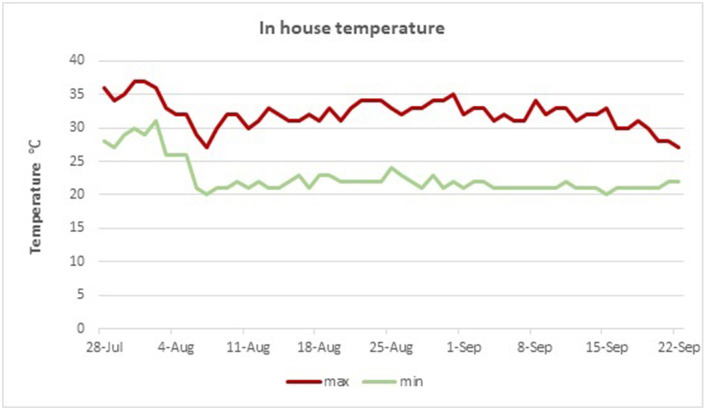
Measured in house minimum and maximum temperatures during the experimental period.

**Figure 2:**
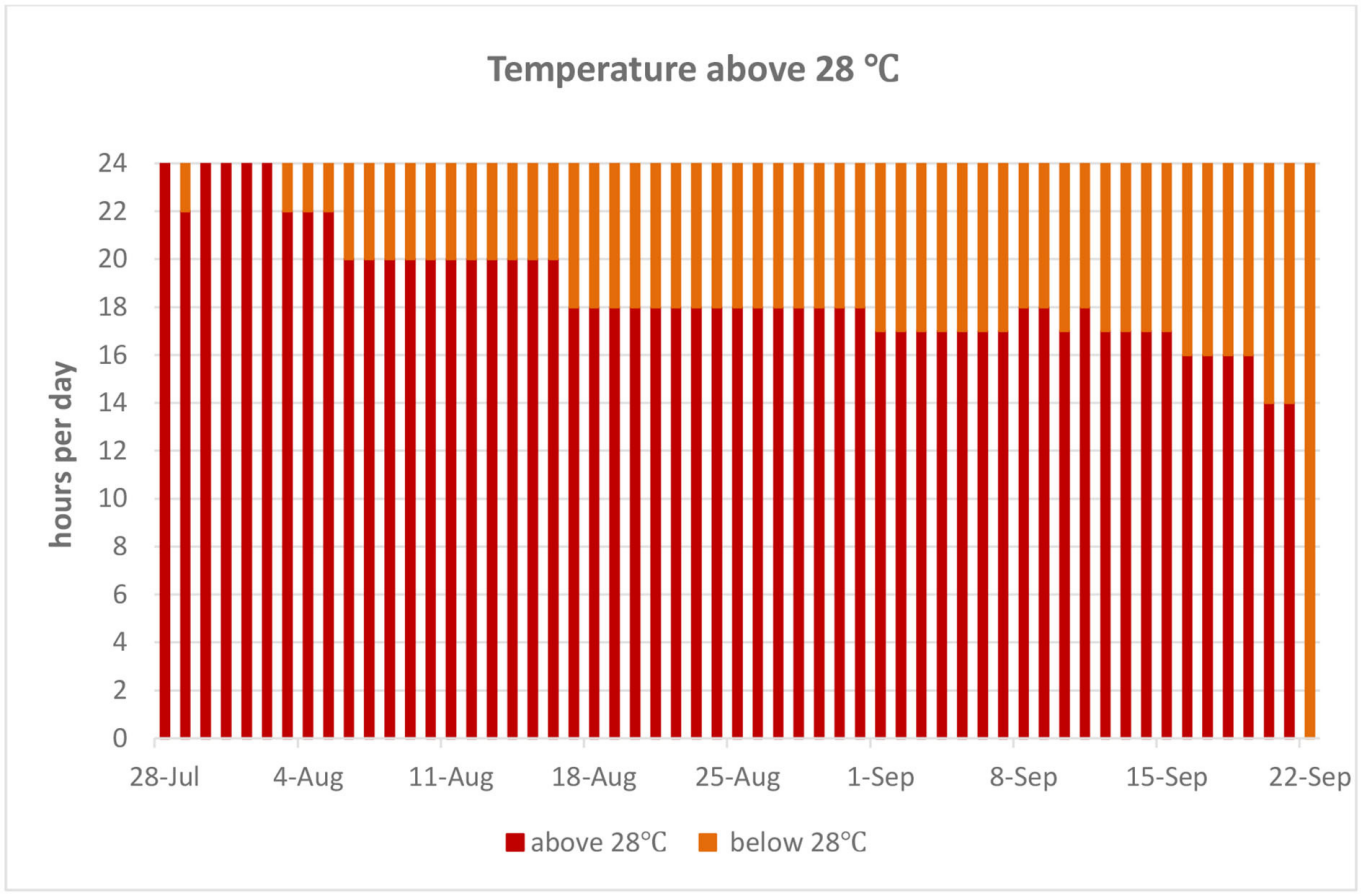
Total daily hours of in-house temperatures exceeding 28°C.

The experiment was conducted between mid-July and mid-September, when the temperature is substantially high during typical Mediterranean summer temperate conditions, in a poultry house without modern ventilation systems. In-house temperatures were recorded during the morning (9:00), local noon (13:00), and evening hours (17:00). Regional temperature data are presented for the period of the trial, and total hours per day with in-house ambient temperatures exceeding 28°C are presented in Figures 1, 2, respectively.

### Determination of the total phenolic content of diets, additives, and egg yolks

PHYTO supplemented diets were analyzed for their total phenolic content (TPC) according to the method of Singleton et al. (22) and expressed as gallic acid equivalents (GAE) mg/g, as determined by using the Folin–Ciocalteu assay. Extraction of phenolics from egg yolks and determination of the total phenolic content were carried out according to the protocol of Shang et al. (23). For the extraction of phenolic compounds, the yolks were separated from the albumen and homogenized. About 8 mL of 50% aqueous methanol [MeOH(aq) 50%] was added to 2 g of homogenized egg yolk, and the mixture was vortexed and then centrifuged at room temperature (3,000 g, 20 min). The supernatant was collected, and 4 mL of it was added to 400 μl TCA 10% w/v. After a second centrifugation (3,000 g, 20 min), the supernatant was once again collected. For the determination of the total phenolic content, 125 μl of egg yolk extract was added to test tubes containing 125 μl of Folin–Ciocalteu reagent and 2.25 ml of Na_2_CO3 7%. The samples were left in the dark for 30 min, at room temperature, and their absorbance was measured afterward, at λ = 725 nm. The results were expressed as μg of gallic acid equivalents/ mL of extract (μg GAE/mL extract).

### Hen performance

Hens were weighed prior to offering the experimental diets and at the end of the trial. Egg production and feed intake were recorded weekly. To provide an overall estimation of feed efficiency during the experimental period, we calculated the average of the egg mass produced by dividing the sum of egg mass at the start and at the end of the experimental period. Then, we calculated the biweekly feed: egg weight ratio for each cage using weekly measured feed intake data. The estimated feed: egg weight ratio is expressed as g of feed per g of eggs produced according to Papadopoulos et al. (24).

### Blood sampling and biochemical analysis

Two hens per cage were randomly selected at the end of the experimental period for blood sampling *via* the brachial vein in tubes without anticoagulant (BD Vacutainer^®^ Plymouth, UK) to obtain serum. The samples were allowed to clot for 4–5 h at 4°C, which were centrifuged at 3,000 RPM, for 15 min (HERMLE, Wehingen, Germany), and the sera were collected and stored in −20°C pending analysis photometrically (Siemens ADVIA 1800 Chemistry System, Erlangen, Germany). Serum samples were tested for antioxidant enzymes, namely alkaline phosphatase (ALP), alanine transaminase (ALT), aspartate transaminase (AST), gamma-glutamyltransferase (γ-GT), and corticosterone (CORT) to estimate the effect of PHYTO supplementation on liver function. Antioxidant enzyme production was assessed by measuring superoxide dismutase (SOD), glutathione peroxidase (GPx), and catalase (CAT) to assess endogenous antioxidant enzyme production by ELISA Kits (Siemens Healthcare GmbH, Erlangen, Germany). Thiobarbituric acid-reactive substance (TBARS) was assessed as a marker of hen oxidative status and corticosterone as a general marker of heat stress by the methodology of Ahn et al. (25).

### Evaluation of liver histopathology and small intestine histomorphometry and histomorphology

One out of two selected hens per cage selected for blood sampling was euthanized at the end of the trial for evaluating liver macroscopic and microscopic observations of HS-related lesions and intestinal histomorphometrical alterations. The liver was dissected and was directly evaluated macroscopically. Then, samples were collected from each liver, fixed in a 10% formalin solution, and embedded in paraffin. Section 4 μm were taken and stained with hematoxylin and eosin (H&E) for histopathological analysis. They were also stained with Martius Scarlet Blue trichrome staining (MSB) for fibrin visualization. At a microscopic level, liver samples were examined for alterations caused by thermal stress, which are related to the presence of hemorrhagic necrosis, microvascular thrombosis, and hepatocellular necrosis (26, 27). Closed intestinal samples were obtained from the duodenum, jejunum (before Meckel's diverticulum), and ileum, submitted to the hemicylindrical section, and were fixed in 10% formaldehyde. From each sample, section 4 μm were taken, routinely processed, and stained with H&E for morphological and morphometrical analysis, using light microscopy, according to the criteria of Gava et al. (28). Only intact villi were measured for villus height (VH) and crypt depth (CD). The measurements were performed with a Nikon microscope (Nikon Eclipse 200, Tokyo, Japan) coupled with a computer-assisted digital image analysis software (Image-Pro Plus, 2017). For the small intestine's morphological evaluation, the Chiu/Park scale was applied (29).

### Egg quality

To determine egg quality indices, four eggs from each replicate (100 eggs per group) were collected during the first and last day of the trial (8th week). Egg quality indices were assessed at the beginning of the trial as a proxy to certify that there were no potential biases related to the level of oxidative stress experienced by the hens prior to their allocation to the treatments. All eggs were weighted using a balance (Navigator TM, N2B110, OHAUS Corporation, city, country). Egg length and width were measured with a digital caliper (EMC, LTD) of 0.01 mm accuracy, while egg shape index was calculated using the formula: shape index = (width/length) × 100. Eggshell color was measured with a reflectometer (EQ Reflectometer, York Electronics Centre), while egg-specific gravity was calculated using the method based on Archimedes' principle. Eggshell deformation was determined by performing a compression test with Texture Analyzer (TA.HD. plus Texture Analyzer, Stable Micro Systems Ltd, Surrey, UK). Eggs were placed horizontally and compressed on the equator under a force of 500 g for 10 s. The distance by which the eggshell deformed was recorded. Eggshell breaking force was also determined with Texture Analyzer (TA.HD. plus Texture Analyzer, Stable Micro Systems Ltd, Surrey, UK) on the equatorial region with a compression platen. The peak force (kg·m/s^2^) on the force–time graph was recorded as the breaking force. Haugh units were measured by using designated equipment by the EQM York Electronics Centre (Egg Quality Microprocessor, Technical Services & Supplies Ltd., Dunnington, York, UK). Egg yolk was separated from the albumen and weighted on a balance (Navigator TM, N2B110, OHAUS Corporation, Nanikon, Switzerland). Yolk color was scored visually by using the Yolk Color Fan^®^ scale and measured instrumentally with Chroma Meter CR-410 (Konica Minolta, Osaka, Japan) using the L^*^a^*^b^*^ color space. Subsequently, the eggshell was washed to remove the adhering albumen and air-dried. The thickness of the eggshell with the membranes was measured with a caliper (AMES, Waltham, MA, USA, accuracy 0.001 in), while its weight was measured using a balance (Navigator TM, N2B110, OHAUS Corporation, Parsippany, NJ, USA). Albumen weight was calculated by subtracting the weights of egg yolk and shell from the weight of the egg.

### Egg yolk oxidative stability of fresh eggs

Eggs collected at the end of the trial were delivered fresh to the Laboratory of Nutrition, School of Veterinary Medicine, Aristotle University, in a cool box at 4°C immediately after collection and tested upon arrival. To determine the oxidative stability of egg yolk, TBARS was measured based on malondialdehyde content, a secondary lipid oxidation product formed by hydrolysis of lipid hydroperoxides, according to the method of Ahn et al. (25). Absorbance was read at 530 nm against a blank sample using an UV-Visible spectrophotometer (UV-1700 PharmaSpec, Shimadzu, Japan). Protein carbonyl determination was Image-Pro Plus, Rockville, USA. Briefly, 50 μL of 20% TCA was added to 50 μL of egg albumen homogenate (diluted 1:2), and this mixture was incubated in an ice bath for 15 min and centrifuged at 15,000 g for 5 min at 4°C. The supernatant was discarded, and 500 μL of 10 mmol/L 2,4-dinitrophenylhydrazine (DNPH; in 2.5 N HCL) for the sample (500 μL of 2.5 N HCL for the blank) was added in the pellet. Calculation of protein carbonyl concentration was based on the molar extinction coefficient of DNPH (22 × 103 M^−1^ cm^−1^).

### Statistical analysis

Data were analyzed using the IBM SPSS Statistics Ver.25 software package (SPSS 25.0 Version, Chicago, IL, USA). Cage was considered as the experimental unit for all data. Statistical significance was considered at *P* < 0.05 and tendency at 0.05 < *P* < 0.1. The results are presented as mean ± standard error of the mean (pooled SEM) unless stated otherwise. Daily performance data were used to calculate ADFI and egg-laying production biweekly and for the overall production period. The estimated feed: egg weight ratio was calculated for the same intervals. Performance data were analyzed with one-way ANOVA of the general linear model. Egg quality data obtained at the start and at the end of the experimental period (1st and 8th week) and serum biochemical measurements were analyzed with one-way ANOVA of the general linear models. Before statistical analysis, Levene's test was applied to test the homogeneity of the variances. Histomorphometrical measurements were compared between treatments using the Mann–Whitney test with the GraphPad Prism software (version 9.1.2 for Windows^®^, GraphPad Software, San Diego, CA, USA). Chi-square test was also performed to investigate the effects on the degree of hepatocellular necrosis, hemorrhagic lesions, and microvascular thrombosis.

## Results

### Determination of the total phenolic content in diets and eggs

The results of the TPC analysis showed that the diet of the control group contained 4,305 mg GAE/g dry mass. Accordingly, the PHYTO diet contained 16.384 mg GAE/g dry mass. The PHYTO product contained 181.2 mg TCP as far as the TPC of egg yolk is concerned, and samples from treated hens showed a substantial increase in the PHYTO group vs. the control group (123.077 μg GAE/mL extract vs. 75.846 ± 9.321 μg GAE/mL extract).

### Hen performance

The effects of the dietary supplementation with PHYTO on laying hen performance parameters are shown in Table 2. Egg production (%) was significantly increased (*P* < 0.05) by PHYTO supplementation during weeks 28–29 and for the overall experimental period and remained within expected levels for the birds' age. ADFI of the PHYTO group was significantly decreased during weeks 26–27 and 28–29 of the trial (*P* = 0.003 and *P* = 0.010, respectively), while it tended to be lower during the overall period (*P* = 0.061). PHYTO supplementation resulted in a numerically lower estimated feed: egg weight ratio, at weeks 26–27 (*P* = 0.024) and 28–29 (*P* = 0.043), compared to the control treatment. Hen BW did not differ significantly, either at the start or at the end of the trial between treatment groups.

**Table 2:** Effect of dietary supplementation with PHYTO on laying hen performance.

	**Groups** ^**1**^			
**Egg production, %**	**Control**	**PHYTO**	**SEM^2^**	***P***
Week 24 (adaptation)	86.4	87.0	0.734	0.680
Weeks 25–26	91.3	92.7	0.601	0.225
Weeks 27–28	86.4^a^	91.5^b^	0.796	**<0.001**
Weeks 29–30	86.6	89.4	0.916	0.145
Weeks 31–32	81.0	84.0	1.500	0.161
Total (25–32 w)	86.8^a^	89.0^b^	0.343	**<0.001**
**Average daily feed intake, g**				
Week 24 (adaptation)	107.46	106.36	0.349	0.118
Weeks 25–26	112.48^b^	110.34^a^	0.377	**0.003**
Weeks 27–28	114.81^b^	112.66^a^	0.426	**0.010**
Weeks 29–30	115.64	115.26	0.300	0.530
Weeks 31–32	118.26	118.08	0.333	0.791
Total (25–32 w)	113.73^x^	112.54^y^	0.160	0.061
**Feed: Egg weight ratio**				
Week 24 (adaptation)	2.00	1.93	0.020	0.087
Weeks 25–26	2.10	2.00	0.020	**0.024**
Weeks 27–28	2.14	2.05	0.023	**0.043**
Weeks 29–30	2.16	2.10	0.021	0.167
Weeks 31–32	2.20	2.15	0.022	0.197
Total (25–32 w)	2.10	2.04	0.020	0.120
**BW (kg)**				
Week 25	1.865	1.864	4.846	0.866
Week 32	1.978	1.981	7.775	0.851

^a, b^Values in the same row with different superscripts differ significantly (P ≤ 0.05).

^x, y^Values in the same row with different superscripts tend to differ (0.05 < P ≤ 0.10).

^1^Groups of layer hens fed the control or the supplemented diet; ^2^SEM, standard error of the mean.

### Blood markers

The effects of the dietary supplementation with PHYTO are presented in Table 3. The level of TBARS, CAT, and ALT was significantly lower in the PHYTO supplemented group than the control one (*P* = 0.021, *P* < 0.001, and *P* = 0.014, respectively). Furthermore, PHYTO supplemented hens showed significantly lower circulating corticosterone levels (*P* < 0.001). On the contrary, the levels of SOD, GPx, AST, and gGT were similar between groups (*P* > 0.05).

**Table 3:** Effect of dietary supplementation with PHYTO on serum biochemical parameters measured in two hens per replicate cage (*n* = 50 per group) at the end of the experimental period (week 32).

	**Groups** ^**1**^			
**Parameter**	**Control**	**Phyto**	**SEM^2^**	***P***
ALP (IU/L)	271.7	344.8	51.58	0.182
ALT (IU/L)	10.6^b^	7.8^a^	0.52	**0.014**
AST (IU/L)	486.1	368.6	47.41	0.366
γ-GT (IU/L)	15.6	15.1	0.886	0.683
TBARS (nmol/ml)	20.77^b^	13.49^a^	1.59	**0.021**
CAT (U/ml)	1.17^b^	0.54^a^	0.077	**<0.001**
SOD (U/ml)	1.36	1.32	0.040	0.645
GPx (nmol/ml)	0.191	0.197	0.015	0.847
Corticosterone μg/dL	0.97^b^	0.75^a^	0.031	**0.001**

ALB, albumin; ALP, alkaline phosphatase; ALT, alanine aminotransferase; AST, aspartate aminotransferase; **γ-**GT, gamma-glutamyltransferase; TBARS, thiobarbituric acid-reactive substances; CAT, catalase; SOD, superoxide dismutase; GPx, glutathione peroxidase (GPx).

^a, b^Values in the same row with different superscripts differ significantly (P ≤ 0.05).

^1^Groups of layer hens fed the control or the supplemented diet; ^2^SEM, standard error of the mean.

### Liver histopathology and small intestine histomorphometry and histomorphology

**Figure 3:**
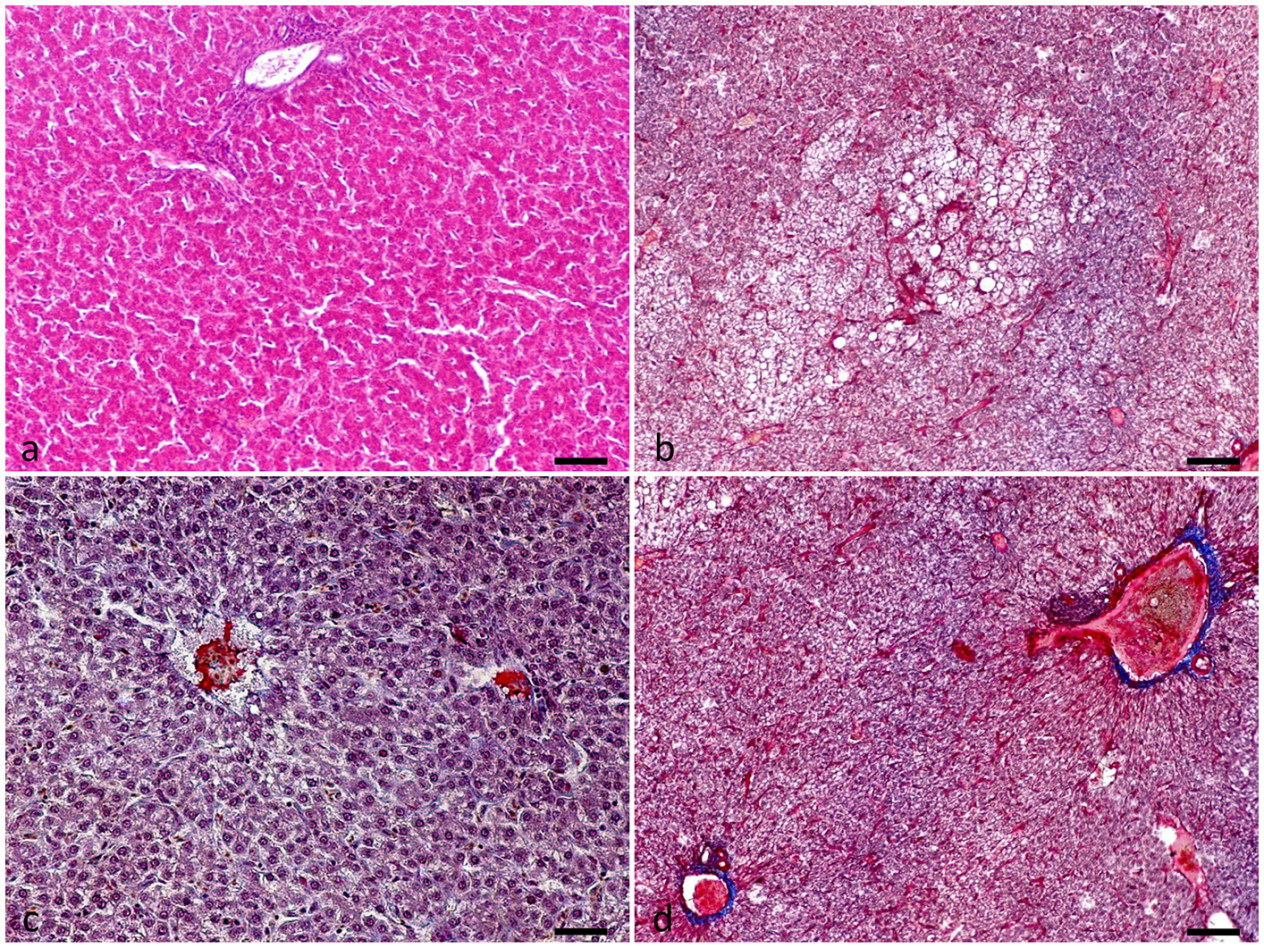
Liver evaluation of hens at the end of the experimental period (week 32). **(a)** Normal liver tissue, **(b–d)** Presence of intravascular organized fibrin consistent with the formation of thrombi, degeneration, and necrosis of hepatic cells with micro-hemorrhages. Hematoxylin and Eosin **(a)**. Martius-Scarlet-Blue **(b–d)**. Scale bar: 250 μm.

**Figure 4:**
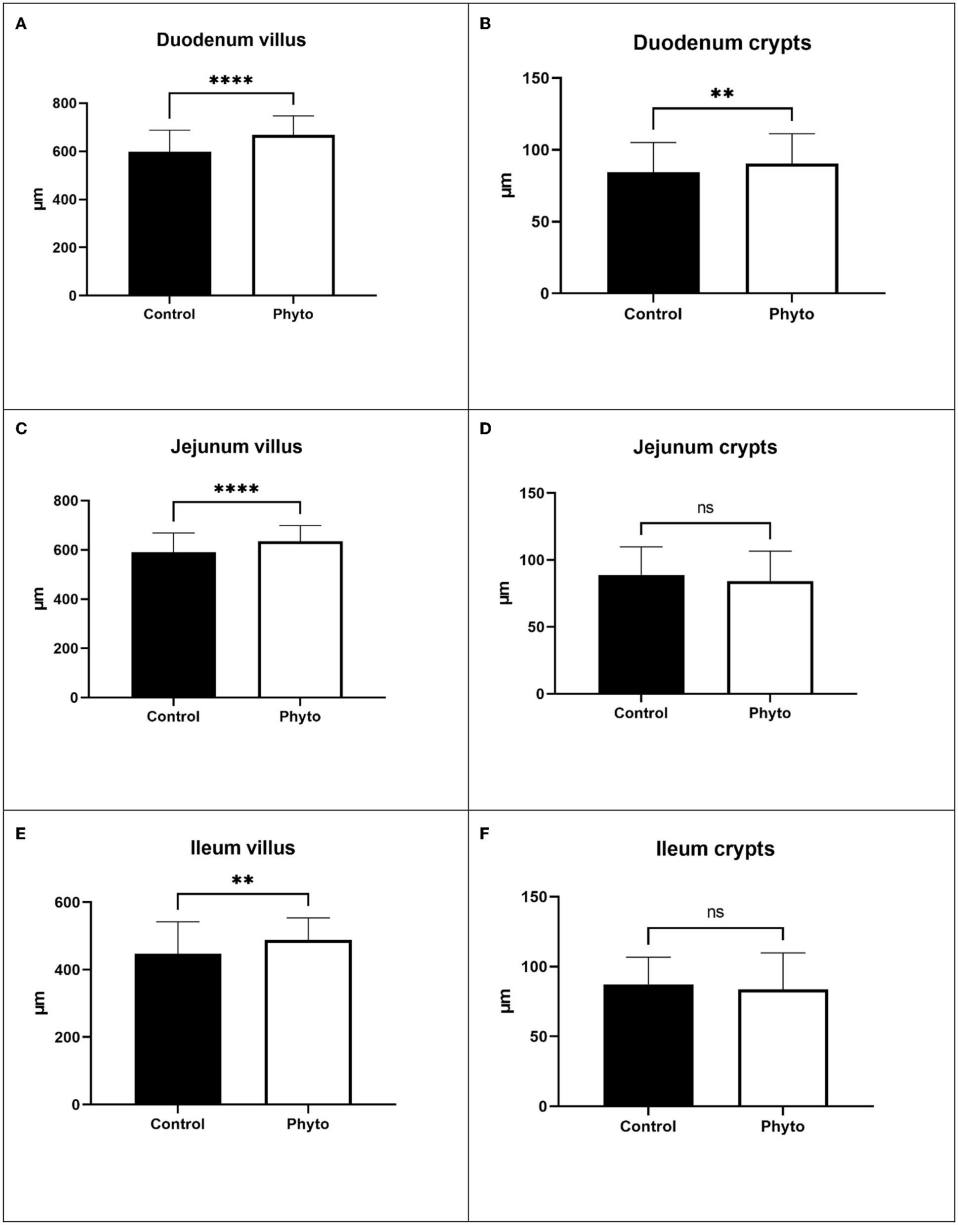
Effect of dietary supplementation with PHYTO on duodenal, jejunal, and ileal villus height and crypt depth of laying hens at the end of the trial (1 hen per replicate cage; *n* = 25 per treatment). **(A)** Duodenum villus; **(B)** Duodenum crypts; **(C)** Jejunum villus; **(D)** Jejunum crypts; **(E)** Ileum villus; **(F)** Ileum crypts. Bars represent mean values and error bars standard deviation of values within each experimental group. ****P < 0.0001; **P < 0.01; ns, not significant.

Liver evaluation (Figure 3) showed that both the incidence and severity of hepatocellular necrosis and microvascular thrombosis were reduced in the PHYTO group in comparison with the control group (*P* = 0.034 and *P* = 0.015, respectively; Table 4). The effects of PHYTO supplementation on intestinal histomorphometrical parameters are presented in Figure 4. VH was significantly higher in the duodenum, jejunum, and ileum in the group supplemented with the phytogenic additive compared to the control one (*P* < 0.0001, *P* < 0.0001, and *P* < 0.01, respectively). CD was also higher in the PHYTO group than the control, however, only in the duodenal part (*P* < 0.01). Mucosal damage was noticed in the duodenum of the control group compared to the PHYTO group. Based on the Chiu/Park scale, the damage was in the subepithelial space with moderate (degree 2) to the massive lifting of villi and partial tip denudation (degree 3). The intestinal segments of the jejunum and ileum were normal or with limited minor injuries (degree 1), and there were no differences among the groups (29, 30).

**Table 4:** Effect of dietary supplementation with PHYTO on hen liver evaluation at the 8th week of the trial.

	Groups*			
**Liver evaluation**	**Control**	**PHYTO**	**SEM**	**P**
Hepatocellular necrosis	1.48^b^	1.36^a^	0.107	**0.034**
Hemorrhagic lesions	0.96	0.60	0.144	0.370
Microvascular thrombosis	1.60^b^	0.84^a^	0.154	**0.015**

^*^N = one liver sample per replication (25 liver samples per group).

^a, b^Values in the same row with different superscripts differ significantly (P ≤ 0.05).

^1^Groups of layer hens fed the control or the supplemented diet; ^2^SEM, standard error of the mean.

### Egg quality and oxidative stability

Egg quality parameters measured in eggs collected at the start and at the end of the trial are presented in Tables 5A,B, respectively. There were no differences between groups on egg quality parameters prior to offering the experimental diets. A tendency for increased eggshell thickness (*P* = 0.07) and a significant increase in resistance to breaking force (*P* = 0.022) and egg diameter (*P* = 0.036) were found for the PHYTO group, compared to the control group, whereas all other parameters remained unaffected (*P* > 0.1). As far as egg oxidative stability is concerned, egg white (albumen) oxidation expressed as protein carbonyls at the 8th week of the trial showed no difference between groups (*P* > 0.1). The egg yolk oxidation test showed that the eggs of the PHYTO group had lower TBARS values (*P* = 0.001) at the end of the experimental period (*P* = 0.027), whereas no differences were noted for eggs collected on the first day of experimentation (*P* > 0.1).

**Table 5A:** Effect of dietary supplementation with PHYTO product on egg quality parameters at the 1st week of the trial.

	Groups*, 1			
**Egg quality parameter**	**Control**	**PHYTO**	**SEM^2^**	***P***
Egg weight (g)	60.82	60.72	0.700	0.944
Yolk weight (g)	16.20	16.03	0.192	0.660
Egg white weight (g)	39.01	39.25	0.664	0.860
Egg weight in water (g)	4.35	4.69	0.102	0.101
Egg weight, specific (g/cm^3^)	1.068	1.065	0.0018	0.101
Haugh unit	77.92	73.56	1.481	0.143
Egg diameter (cm)	43.44	43.36	0.200	0.836
Egg lateral index (cm)	59.07	59.42	0.452	0.705
Egg shape index	73.64	73.16	0.477	0.625
Egg shell thickness (mm)	0.45	0.43	0.006	0.606
Egg shell weight (g)	5.61	5.45	0.104	0.433
Egg yolk color	14.08	13.69	0.116	0.136
Egg shell color	33.14	27.03	1.997	0.127
**Egg strength**				
Eggshell breaking force (N/m^2^)	3750.2	3706.9	0.160	0.606
Eggshell deformation (N/m^2^)	0.029^x^	0.024^y^	0.001	0.084
**Egg Yolk TBARS**				
TBARS, nmol/mL	1.709	1.871	0.038	0.973

^*^N = 25 (pens), four eggs per replication.

^x, y^Values in the same row with different superscripts tend to differ (0.05 < P ≤ 0.10).

**Table 5B:** Effect of dietary supplementation with PHYTO on egg quality parameters at the 8th week of the trial.

	Groups*, 1			
**Egg quality parameter**	**Control**	**PHYTO**	**SEM^2^**	***P***
Egg weight (g)	65.48	67.12	0.625	0.194
Yolk weight (g)	16.37	16.78	0.156	0.194
Egg white weight (g)	43.41	44.50	0.414	0.194
Egg weight in water (g)	4.93	5.11	0.079	0.261
Egg weight, specific (g/cm^3^)	1.082	1.083	0.001	0.791
Haugh unit	85.15	89.29	1.363	0.131
Egg diameter (cm)	44.00^a^	44.78^b^	0.187	**0.036**
Egg lateral index (cm)	60.24	60.02	0.431	0.788
Egg shape index	73.21	74.73	0.557	0.174
Egg shell thickness (mm)	0.45^x^	0.48^y^	0.007	0.070
Egg shell weight (g)	5.69	5.83	0.054	0.194
Egg yolk color	13.60	13.68	0.156	0.800
Egg shell color	27.71	25.58	1.275	0.409
**Egg Strength**				
Eggshell breaking force (N/m^2^)	3706.96^a^	4250.16^b^	119.83	**0.022**
Eggshell deformation (N/m^2^)	0.030	0.031	0.0015	0.783
**Egg Yolk TBARS**				
TBARS, nmol/mL	3.003^b^	2.089^a^	0.101	**0.001**
**Egg protein carbonyls**				
Albumen protein carbonyls, nmol/mL (day 1)	56.33	53.19	2.705	0.567

^*^N = 25 (pens), four eggs per replication.

^a, b^Values in the same row with different superscripts differ significantly (P ≤ 0.05).

^x, y^Values in the same row with different superscripts tend to differ (0.05 < P ≤ 0.10).

^1^Groups of layer hens fed the control or the supplemented diet; ^2^SEM, standard error of the mean.

## Discussion

In the present trial, we investigated the effects of supplementing phytonutrient solution consisting of a plant extract combination of Curcuma and Scutellaria in young laying hens raised in typical high ambient temperature conditions prevailing during the summer period in the Mediterranean basin. The temperature threshold at which HS occurs may vary as hen thermotolerance is affected by bird characteristics such as age, genotype, and level of productivity. In addition, HS parameters such as intensity, duration, whether it is cyclic or constant, and relative humidity levels define the magnitude of the effects on laying hen performance. Regardless, measured in-house temperatures exceeded 28°C which persisted for more than 8 h per day for the overall trial period; therefore, it is reasonable to state that hens were experiencing HS (31). Since the trial started in July, hens were already exposed to elevated temperatures typical of the Mediterranean climate prior to supplementation, although in-house temperatures were not recorded during that period. It has been clearly demonstrated that exposure to heat stress at peak production either cyclic or constant may lead to decreases in BW gain (32). Since layers did not lose BW between the start and the end of the trial, one can assume that they partially adapted to it (4), and/or the heat stress was of mild intensity (4, 32).

It was expected that under such challenging environmental conditions, mitigating oxidative stress and ensuing inflammation would ameliorate performance. Indeed, in response to the PHYTO supplementation, egg production was increased, and this was particularly evident between 28 and 29 weeks of age. Supplemented hens maintained an overall higher rate of egg production which was accompanied by a decreased ADFI over the period 26–29 weeks and consequently improved the estimated feed: egg weight ratio. Although ADFI reduction is the primary driver for the impaired performance observed under HS conditions in laying hens (4, 32), it is apparent that this plant extract association acted mainly on egg production efficiency. An absence of effect on ADFI is in agreement with the results of studies investigating curcumin supplementation in heat-stressed laying hens (14) and SCUT supplementation in hens raised in normal ambient conditions (17).

We hypothesized that positive effects of the plant extract supplementation on performance will be reflected on markers of systemic oxidative stress, but also on heat stress sensitive tissues such as the liver (33, 34) and the intestine (12, 35), and on animal products such as the egg (36). Positive effects of dietary supplementation with CUR extracts and its principal metabolite curcumin have been previously observed in poultry species in the absence of HS (37), but also specifically in heat-stressed laying hens (13, 14). On the contrary, there are no studies investigating the effects of offering SCUT, or its active metabolites in heat-stressed laying hens although positive effects on laying hens, broilers, and other livestock species have been previously observed (16 for a recent review). Although dietary supplementation of a combination of CUR and SCUT, or their PSMs show synergistic effects in comparison with their isolated supplementation (18, 38), the underlying mechanisms have not yet been fully elucidated.

The combination of morphological and morphometrical analyses allows a reliable evaluation of subtle differences in intestine and the effects of dietary intervention strategies under HS conditions (30). It is well-established that HS can negatively impact intestinal histomorphometric features, leading to impaired nutrient digestion and absorption (39). Recent studies have shown that HS reduces nutrient digestibility (3) and absorption capacity as indicated by decreased VL and increased CD in laying hens (40) and causes a downregulation of nutrient transporters in the intestinal tract of broiler chicken (41). In our study, the effects on egg production were accompanied by increased villosity across the gastrointestinal tract. The morphological analysis indicated that the duodenum was mainly affected by HS. Jejunum and ileum morphology were unaffected. In a previous study, duodenum and jejunum morphology showed alterations upon HS exposure, whereas the ileum remained undamaged by HS (30). Amelioration of intestinal indices was also observed in laying hens offered CUR powder and raised in thermoneutral conditions (42), which were accompanied by decreased ileal *Escherichia coli* populations. A decreased microbial content in the cecum has also been found in response to SCUT supplementation in laying hens (17). Upon oral intake, curcumin is effectively taken up by the intestinal epithelial cells, where they initiate or modulate several signaling pathways that ultimately lead to downregulation of inflammatory pathways preventing the disruption of intestinal barrier function (43, 44). It has been proposed that the resulting attenuation of luminal bacteria or bacterial products such as LPS underlies the observed beneficial effects on intestinal function (45). As far as baicalin is concerned, which is the principal active component of SCUT extracts, a downregulation of intestinal inflammation and associated oxidative stress in deoxynivalenol (DON)-treated piglets, in response to its dietary supplementation, has been observed (46). Both plant extract principal constituents have been shown to inhibit NF-κB and to increase mTOR signaling to modulate downstream inflammatory and oxidative responses in the intestine (43, 46). Importantly, although offering CUR extract alone was not enough to decrease gut inflammation induced by HS, a mixture of CUR and SCUT extracts decreased gut inflammation induced by heat, or *S. Enteritidis* infection in broiler chicks (18). Since, microbiota composition is modulated by dietary intake of PSMs contained in both extracts (44, 47) and given that HS alters fecal volatile fatty acid production and nutrient digestibility in laying hens (3), further research is required to disassociate their effects on laying hen microbiota composition.

Liver evaluation for hens kept under HS showed that the plant extract constituents positively affected the integrity of hepatic and endothelial cells, resulting in milder hepatocellular necrosis and microvascular thrombosis. In general, the pathogenesis of HS on liver tissue injury is multifactorial (34). To some degree, it may be affected by increased bacterial translocation and their toxins from the intestinal tract which consequently induces an inflammatory response (48). It is suggested that HS affects initially the endothelial cells, which leads to diffuse vascular damage and activation of the coagulation cascade, resulting in hypercoagulability, the formation of multiple microthrombi, and, subsequently, diffuse microvascular thrombosis (49). Main lesions observed are the multifocal to diffuse bleeding, hemorrhagic necrosis, and widespread microthrombi due to indirect endothelial damage and direct hyperthermic hepatocellular damage (50). In agreement with liver histological findings, the reduced degree of liver injury was accompanied by a significant reduction in ALT in PHYTO supplemented hens. An increase in ALT has been previously attributed to cellular leakage and loss of the functional integrity of hepatic cell membrane induced by HS, resulting in its release from the cytoplasm (51). Both CUR and SCUT and their metabolites possess hepatoprotective effects. Recently, curcumin supplementation reduced inflammatory cell infiltration around the central veins, dilation of sinusoidal capillaries, dilation of central veins, reduced the size of hepatocytes, and necrosis in the hepatic lobules in heat-stressed laying hens. This was associated with a downregulation of pro-inflammatory cytokine gene expression levels and protein expression of NF-κB in the liver. These effects may be related to the reduced translocation of intestinal endotoxin LPS in the circulation, which activates toll-like receptor signaling pathways to induce Nf-kB inflammatory pathways in the liver (52). On the contrary, recent studies highlight the effects of baicalin in attenuating inflammatory pathways under conditions which challenge liver health and function (53, 54). Interestingly, the combination of curcumin and baicalin has been recently shown to exert superior hepatoprotective effects in ethanol-challenged rats, in comparison with monotherapy with the respective metabolites (38), set aside already demonstrated the effects on the intestine (18). A synergistic activity in the liver has been loosely attributed to their combined effects on multiple signaling pathways regulating the pro-inflammatory response and transcription factors, although further research is required (38).

In line with an improvement of gut morphology and liver integrity, a reduction in corticosterone was observed, which is typically elevated in response to stressors, among HS. Briefly, HS exposure leads to the activation of the neuroendocrine system, which stimulates the hypothalamic–pituitary–adrenal axis (HPA) to increase plasma corticosterone (55). Both baicalin and curcumin are viewed as potent anti-stress agents, capable of modulating the HPA axis (56, 57). Offering curcumin has been recently shown to reduce laying hen corticosterone levels in heat stress conditions (14), while SCUT extract has been shown to modify the thermoregulatory behavior of broilers raised in moderate HS conditions (58). Corticosterone has pro-oxidative functions leading to increased mitochondrial metabolism (59), increases energy deposition at the expense of reproductive function (60), and may lead to decreased steroidogenesis and as a result impaired ovarian development and function (61, 62). Therefore, positive effects observed on egg production of supplemented birds may be associated with reduced corticosterone production.

Laying hens supplemented with the plant extract combination experienced systemic oxidative stress to a smaller degree as indicated by blood TBARS values, according to expectations, however, that occurred without a change in systemic levels of serum antioxidant enzymes, apart from CAT, which was in fact reduced in supplemented bird's contrary to conventional expectations. Antioxidant enzyme activities are believed to be compromised by heat stress conditions corroborated with a reduction in Nrf2 expression, although differences in antioxidant enzyme activities might largely depend on the HS conditions, species, tissue, and sampling in relation to the induction of HS (8, 10). On the contrary, the effects of PSMs are partially attributed to their ability to upregulate transcription factor Nrf2-mediated antioxidant enzymes such as CAT, GPx, and SOD (9). Previous studies in heat-stressed layers offered curcumin have illustrated that CAT concentration was increased following 3 and 6 weeks (13) and 6 weeks of HS exposure (14) in comparison with non-supplemented birds. Since in this study supplemented hens were experiencing oxidative stress to a smaller degree, a lower requirement for CAT production may be implied. Future trials should focus on laying hen oxidative responses over time under varying environmental conditions in response to plant extract supplementation.

As far as egg quality is concerned, egg weight, eggshell thickness, eggshell percent, and eggshell density are negatively affected by high ambient temperature (63). Other egg quality parameters, like eggshell color, are mostly affected by genetic factors and to a lesser extent by other factors such as age, diseases and stress, electrolyte balance, and nutrient levels. In our study, the eggshell and egg yolk color were not substantially influenced PHYTO supplementation. Supplementation with CUR or curcumin has been previously shown to improve eggshell thickness, eggshell strength, and albumen height (14) and parameters, such as specific gravity and yolk color (64). Importantly, these effects may be dose- and time-dependent in relation to sampling following supplementation (42). There is a scarcity of studies on the effects of SCUT supplementation on egg quality. Although there was no effect on albumen height in the present trial, it has been previously shown that eggs produced by SCUT supplemented hens showed a tendency to have greater Haugh units after 2-week storage (17).

Our results showed that enrichment of the diet with a PHYTO product bearing a substantial TCP content for 56 days improved substantially yolk oxidative stability in line with an improved hen oxidative status, while albumen protein oxidation was not affected. It has been previously shown that the dietary supplementation with curcumin-laying hens reduced the levels of TBARS in the yolk of fresh and stored eggs (15). Similarly, the MDA contents in stored eggs were significantly lowered by feeding SCUT extract (17). These results collectively show that SCUT and CUR and/or its metabolites may minimize lipid peroxidation in stored eggs. It has been reported that the total phenolic content of the diet is well-correlated with oxidative status of the layer hens and the produced eggs (65) or broiler chickens and breast and leg filets (66, 67). However, published information on the beneficial effects of phytonutrients on layers and egg characteristics is considerably less abundant (68). It is well-known that the fat-soluble vitamin content of egg yolk, such as tocopherol or xanthophyll, can be affected by manipulating their dietary levels (69). The literature is prolific with evidence of *in vitro* antioxidant activity of medicinal plants and their extracts, based on their ability to donate a hydrogen or electron, as well as to delocalize the unpaired electron within the aromatic structure protecting biological molecules against oxidation (70), which is well-correlated with their total phenolic content (71). Many published studies have shown an increased postprandial antioxidant capacity of phenolic compounds from various feedstuffs (72, 73); however, when biomarkers of the redox status are measured after phenolic compound consumption, the results obtained are often contradictory (74, 75). In the case of curcumin upon oral intake, it undergoes rapid metabolic redaction and conjugation to glucuronides, resulting in limited systemic bioavailability after oral administration (76). Following an oral dose of 0.1 g/kg administered to mice, a yield of a peak plasma-free curcumin concentration was only 2.25 μg/mL (77). Moreover, it has been previously shown that curcumin is not deposited inside the eggs (15). Similarly, pharmacokinetics of SCUT metabolites, after entering the body, are mostly metabolized in glucuronidase and sulfatase forms (77). Therefore, observed effects may be related to direct or indirect transference of antioxidant compounds to the yolk, since overall oxidative stress experienced by the hens was reduced in this study. Probably, these antioxidants are involved on the reduction in yolk lipid peroxidation, since the TBARS values were reduced.

## Conclusion

In conclusion, offering an additive containing SCUT and CUR extracts increased egg production in laying hens raised in high ambient conditions. The improved performance was accompanied by increased intestinal villosity and reduced liver damage, the effects thought to be mediated by a reduction in oxidative stress and an improvement of intestinal and liver function. Importantly, these effects translated into the production of eggs with improved oxidative stability and eggshell breaking force. Thus, the dietary supplementation of the current PHYTO mixture could be considered as a useful natural alternative to help sustain egg production of layers raised under HS. Future studies should investigate the efficacy of this plant extract combination over time and explore microbiota-related effects.

## Data availability statement

The raw data supporting the conclusions of this article will be made available by the authors, without undue reservation.

## Ethics statement

The trial protocol was authorized by the Research Committee of Aristotle University, Thessaloniki, Greece (number 71553, 28.07.2020). Husbandry, euthanasia, experimental procedures, and biosecurity precautions were conducted in accordance with all welfare requirements described by Good Farming Practice Guidelines (Directive 2010/63/EC; Commission recommendation 2007/526/EC) and were approved by the Research Ethics Committee of the Aristotle University of Thessaloniki.

## Author contributions

IG, PS, and GAP contributed to conception and design of the study. SD and IS organized the database. GAP performed the statistical analysis. IG and PS wrote the first draft of the manuscript. IS, GAP, SD, and TP performed the analysis. IM, VT, IG, and SD performed the experimental trial. All authors wrote sections of the manuscript and contributed to manuscript revision, read, and approved the submitted version.

## Funding

This work was supported by DELTAVIT—GROUPE CCPA, Research Project Code 71553 (Research Committee of Aristotle University of Thessaloniki, Greece).

## Conflict of interest

The use of Scutellaria extract in animal feed is a subject of French patent application FR 14/51501 and PCT application PCT/FR2015/050450.

Authors PS, MP, and FR were employed by CCPA Group.

The remaining authors declares that the research was conducted in the absence of any commercial or financial relationships that could be construed as a potential conflict of interest.

## Publisher's note

All claims expressed in this article are solely those of the authors and do not necessarily represent those of their affiliated organizations, or those of the publisher, the editors and the reviewers. Any product that may be evaluated in this article, or claim that may be made by its manufacturer, is not guaranteed or endorsed by the publisher.
